# Formalization, Annotation and Analysis of Diverse Drug and Probe Screening Assay Datasets Using the BioAssay Ontology (BAO)

**DOI:** 10.1371/journal.pone.0049198

**Published:** 2012-11-14

**Authors:** Uma D. Vempati, Magdalena J. Przydzial, Caty Chung, Saminda Abeyruwan, Ahsan Mir, Kunie Sakurai, Ubbo Visser, Vance P. Lemmon, Stephan C. Schürer

**Affiliations:** 1 Center for Computational Science, University of Miami, Miami, Florida, United States of America; 2 Department of Computer Science, University of Miami, Miami, Florida, United States of America; 3 The Miami Project to Cure Paralysis, Department of Neurological Surgery, University of Miami, Miami, Florida, United States of America; 4 Department of Molecular and Cellular Pharmacology, University of Miami, Miami, Florida, United States of America; Royal College of Surgeons, Ireland

## Abstract

Huge amounts of high-throughput screening (HTS) data for probe and drug development projects are being generated in the pharmaceutical industry and more recently in the public sector. The resulting experimental datasets are increasingly being disseminated via publically accessible repositories. However, existing repositories lack sufficient metadata to describe the experiments and are often difficult to navigate by non-experts. The lack of standardized descriptions and semantics of biological assays and screening results hinder targeted data retrieval, integration, aggregation, and analyses across different HTS datasets, for example to infer mechanisms of action of small molecule perturbagens. To address these limitations, we created the BioAssay Ontology (BAO). BAO has been developed with a focus on data integration and analysis enabling the classification of assays and screening results by concepts that relate to format, assay design, technology, target, and endpoint. Previously, we reported on the higher-level design of BAO and on the semantic querying capabilities offered by the ontology-indexed triple store of HTS data. Here, we report on our detailed design, annotation pipeline, substantially enlarged annotation knowledgebase, and analysis results. We used BAO to annotate assays from the largest public HTS data repository, PubChem, and demonstrate its utility to categorize and analyze diverse HTS results from numerous experiments. BAO is publically available from the NCBO BioPortal at http://bioportal.bioontology.org/ontologies/1533. BAO provides controlled terminology and uniform scope to report probe and drug discovery screening assays and results. BAO leverages description logic to formalize the domain knowledge and facilitate the semantic integration with diverse other resources. As a consequence, BAO offers the potential to infer new knowledge from a corpus of assay results, for example molecular mechanisms of action of perturbagens.

## Introduction

High-throughput screening (HTS) has become the most common approach to identify starting points for the development of novel drugs [1]. Increasingly complex biological systems and processes can be interrogated using HTS, leveraging innovative assay designs and new detection technologies. The establishment of publicly funded screening centers has led to the production and public dissemination of large amounts of HTS data. The Molecular Libraries Probe Production Centers Network (MLPCN), which is part of the NIH Molecular Libraries initiative, offers researchers “access to the large-scale screening capacity, along with medicinal chemistry and informatics necessary to identify chemical probes to study the functions of genes, cells, and biochemical pathways” [2]. MLPCN centers have deposited over four thousand HTS assays testing the effects of several hundred thousand compounds in PubChem [3]. PubChem also contains assay data from non-MLPCN screening centers and research groups. An example of a very recent large-scale public screening effort is the NIH Library of Integrated Network-based Cellular Signatures (LINCS) program, which aims to develop a library of molecular signatures based on gene expression and other cellular changes in response to perturbing agents across a variety of cell types using various high-throughput screening approaches [4]. Other public resources to access screening data include ChEMBL, a database that contains structure-activity relationship (SAR) data curated from the medicinal chemistry literature [5] and the Psychoactive Drug Screening Program (PDSP), which generates data from screening novel psychoactive compounds for pharmacological activity [6]. The EU Open Screen initiative is creating a distributed research infrastructure that involves Europe's leading compound screening sites open to external users and covers numerous technologies and resources required for the discovery of biologically active substances [7]. In addition, private resources, such as Collaborative Drug Discovery (CDD) [8], also make large screening datasets publicly accessible.

Bioassay and HTS results are being submitted to repositories at a fast pace, suggesting that the scope of possible assay formats and technologies has only begun to be explored [9]. Despite being publically available, considerable bioinformatics expertise and specialized software tools are almost always required to extract relevant HTS data, to integrate with other relevant information, and to perform analyses. In fact, resources required for data integration and analysis today routinely exceed those for data production in the first place [10]. Due to lack of uniformity and comprehensiveness of metadata, many repositories are not being utilized to their fullest potential. For example, bioassays in PubChem lack standards to report the HTS results (endpoints), which hinders data integration and analyses [11]. It is currently not possible without considerable curation effort, to identify related assays, for example, those based on the same design (assay principle), the same detection technology, or interrogate protein targets from the same family or in the same pathway. Due to non-uniform reporting of bioactivity and screening endpoints, it is difficult to compare the activity of compounds across different assays.

During the last 10 years, tremendous progress has been made in developing Semantic Web [12] technologies with the goals being the formalization of knowledge, linking information across different domains, and integrating highly complex, diverse, and large datasets. Semantic Web technologies support semantically rich knowledge representations and can solve many data integration problems by linking resources, tracking provenance, and enabling semantic querying [13].

Ontologies have traditionally been used in biological and medical sciences to organize information within a domain and, to a lesser extent, to annotate experimental data. A successful and highly-utilized biomedical ontology is the Gene Ontology (GO) [14], which consists of terminology to describe gene product localization, molecular function, and biological processes. In addition, several hundred ontologies are hosted by the Open Biological and Biomedical Ontologies (OBO) Foundry [15] and the National Center for Biomedical Ontologies (NCBO) [16], [17]. Most of the existing biomedical ontologies describe a specific domain. However, there does not exist an ontology that can be used to describe chemical biology screening assays and data of the type deposited in PubChem. The lack of standards and formalization to describe chemical biology screening assays hinders the full utilization of available data in the domain of drug and probe discovery. To tackle this challenge we developed the BioAssay Ontology (BAO).

The extent to which existing ontologies are formally mapped to each other is limited and in most cases based on language terms [18], rather than formal semantics. One of the reasons for this is that many ontologies have not yet made use of available description logic (DL) features of OWL (Web Ontology Language). The lack of semantics in many biomedical ontologies limits the use of novel Semantic Web technologies to link, access, integrate, infer, and ultimately analyze large-scale cross-domain biological data and also results in misinterpretations [19]. BAO uses OWL 2.0 [20] to define the semantics of the various classes. BAO was designed to enable categorization of assays by concepts that are relevant to interpret screening results and to enable meaningful data retrieval and analysis across diverse HTS assays. BAO is analysis-focused to facilitate conclusions about the molecular mechanisms of action of small molecules based on a large number of result sets; it can thus significantly increase the value of the available aggregated HTS data to the chemical biology, screening and cheminformatics communities.

In BAO, we incorporated information from several of the existing biomedical ontologies, as described in the results section. However, the majority of BAO classes and relationships were newly developed, because existing ontologies, such as the Ontology for Biomedical Investigation (OBI) [21] lack many concepts required to model chemical biology screening data, such as assay design (e.g. viability vs. enzyme reporter), detection technologies (e.g. fluorescent vs. label-free), standardized result endpoints (e.g., IC_50_ vs. percent inhibition), HTS platforms, and detailed bioassay specifications. In another example, the Ontology for Drug Discovery Investigations (DDI) [22] describes aspects of drug discovery, but its scope does not include detailed descriptions of biological assays including assay design, biological target, detection technologies, endpoints, etc. In conclusion, several existing biomedical ontologies include useful terms, but none are applicable to comprehensively describe HTS assays and results and to formalize the domain and scope of chemical biology probe and drug screening. None of the existing ontologies include description of compound activity or endpoint and are therefore not applicable to develop software systems to retrieve biological activities in the context of other assay concepts, such as the biological target or assay design. By creating the necessary relationships among the BAO class hierarchies to describe assays and screening results, we are opening a possibility to query and explore HTS and chemical biology data from various perspectives.

Previously, we reported on the higher-level design of the BioAssay Ontology (BAO) and on the semantic querying capabilities offered by the ontology-indexed triple store of HTS data. Here, we report on our detailed design (major components, subsumption hierarchies, and specification classes of BAO), annotation pipeline, substantially enlarged annotation knowledgebase, and analysis results that have been obtained using that technology. We demonstrate the applications of BAO to annotate assays and screening results as well as entire screening campaigns/projects from PubChem. These annotations enable data retrieval, integration and meta-analyses. We present an analysis of the distribution of over 900 curated assays by major categories of a large set of assays from PubChem and we illustrate how a large number of assays are connected into screening campaigns/projects via assay relationships. To facilitate assay annotations, we developed an Excel-based template based on BAO terminology. A Semantic Web software application, BAOSearch further demonstrates the applicability and value of the ontology to retrieve and analyze diverse assays and screening results. BAO is publically available from our website [23] and the NCBO BioPortal [24].

## Methods

In the following, we use ‘single quotes’ to denote BAO classes and *‘italic font’* to denote the relationships in BAO.

### Ontology development

BAO was constructed using Protégé version 4.1 [25] in OWL (Web Ontology Language) 2.0 [26]. A number of available plugins were used throughout the development process including OWLViz2 [27] and OntoGraf [28] for visualization and DL reasoning engines HermiT [29] and Pellet [30]. Our ontology development follows established ontology engineering methodologies using a combination of top-down, domain expert-driven and bottom-up, data-driven approaches [31]. Several domain experts (biologists and HTS experts) and knowledge modeling experts (DL, OWL) worked together in the development of BAO. Numerous development cycles lead to the current version. BAO was developed in parallel to the manual curation and annotation of a large number of assays from PubChem (vide infra); thus both of these efforts informed each other in a synergistic development cycle.

### PubChem assay annotation

Assay annotation was an iterative manual process performed by several PhD curators. To aid in manual annotation, assays were first clustered based on textual descriptions [32]. Using the terminology from BAO, PubChem bioassays were annotated with ∼100 BAO classes and data properties. BAO terms fall into the main BAO categories ‘assay format’, ‘assay design’, ‘detection technology’, ‘meta target’, ‘perturbagen’, and ‘endpoint’. Assays were grouped by screening campaigns and organized by an ‘assay stage’ (e.g. ‘primary’, ‘secondary’, etc.), ‘assay measurement throughput quality’ (e.g. ‘single concentration single measurement’, ‘concentration response multiple replicates’, etc.), and assay relationships (e.g. *‘is confirmatory assay of’* or *‘is counter assay of’*, etc.), among many others. We also standardized assay ‘endpoints’, e.g.: ‘IC_50_’, ‘EC_50_’, ‘percent inhibition’, etc. The annotations from each assay were populated in a spreadsheet, reviewed by another curator, cross-checked against BAO terms, and then loaded into an RDF (Resource Description Framework) triple store.

### Creation of BioAssay Annotation Tool

The first step in generating the annotation template was flattening the BAO hierarchy manually. The resulting “flat” version of BAO includes all the leaf nodes of BAO with a label/name that reflects the original hierarchical structure. This file was then used in RightField [33], an open-source tool for adding ontology terms to Excel spreadsheets, to generate the BioAssay Annotation template (BAT) by mapping BAO terms to each field [34].

### Integrating external ontologies

We have used standard practices that allow modular reuse of external ontologies namely, Gene Ontology (GO), NCBI organismal classification (NCBITaxon), Cell Line Ontology (CLO), Units of Measurement (UO), and Ontology for Biomedical Investigations (OBI). In order to do this we have used two methods: OntoFox [35] and the module and axioms extraction facility built into the OWL3 API [36]. The former method was used for modular reuse of GO and NCBITaxon ontologies, while the latter method is used for modular reuse of CLO, UO and OBI ontologies. We have added the additional general concept inclusion axioms (GCIs) to BAO to bridge the host concepts and the imported axioms, and we checked the safety and the satisfiability of the knowledge base using the HermiT reasoner.

### Data analyses

The annotated data was analyzed and visualized with Spotfire DecisionSite (http://spotfire.tibco.com/discover-spotfire). Annotated data in an Excel file was first loaded into Spotfire for analysis. AID/assay counts (y-axis) were queried against BAO classes (x-axis) and subcategorized by ‘Assay Stage’. The network visualization of assays and screening campaigns was generated using Cytoscape 2.8.3 (http://www.cytoscape.org/). Final visualizations were exported to PowerPoint for further annotation.

## Results

**Figure 1:**
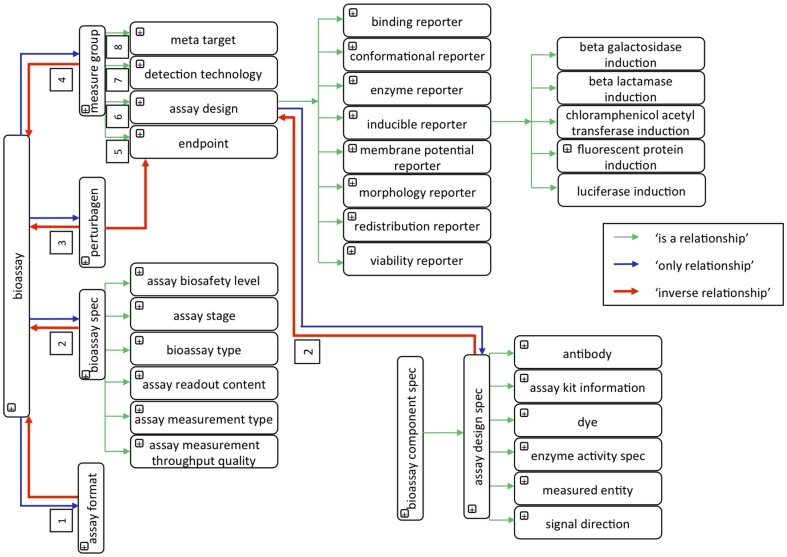
Illustration of the major bioassay components, corresponding specifications, and their relations. Approximation of the BAO logical model. The relationships depicted by blue arrows are as follows: 1) ‘has assay format’, 2) ‘has specification’, 3) ‘has perturbagen’, and 4) ‘has measure group’, and those by red arrows are as follows: 1) ‘is assay format of’, 2) ‘is specification of’, 3) ‘is perturbagen of’, and 4) ‘is measure group of’.

BAO formally describes perturbation bioassays, such as small molecule HTS assays, for the purpose of categorizing the assays and the results by concepts that relate to the screening format, design, technology, target, and endpoints and which are essential to interpret screening results in the context of a molecular mechanism of action. BAO is therefore organized by several major categories, which include multiple levels of subclasses and specifications (Figure 1). A number of specific object property relationships were created to connect the classes and develop a knowledge representation of the biological assays and screening outcomes.

### Salient features of BAO

BAO is instantiated in a well-specified syntax and designed to share a common space of identifiers. The ontology has a formally specified and clearly delineated content. All terms in the ontology also have textual definitions. The high level design of BAO contains the root-level classes (described below), general bioassay specifications, and some of their relationships. ‘Measure group’ is a class created to group experimental outcomes into result sets and thus enables the modeling of multiplexed and multi-parametric assays. Each BAO component includes multiple levels of sub-classes and specification classes that are linked via object property relationships to form a knowledge representation (Figure 1). Specification classes contain additional details or attributes corresponding to the main BAO components.

### Detailed description of BAO main components

BAO has been expanded and refined in several releases available from our website [23]. The most recent release is BAO v1.6. The conceptual structure of BAO was partially described in a previous communication [37]; here we report details of the main BAO classes and describe how they are used in specific assay examples (vide infra). Assay format is a conceptualization of assays based on the biological and/or chemical features of the experimental system. For example, assay formats include biochemical assays - referring to assays with purified protein, cell-based - referring to assays in whole cells, or organism-based - referring to assays performed in an organism. Further details are captured as ‘format specifications’.

‘Assay design’ describes the methodology and implementation of how the perturbation of the biological system is translated into a detectable signal. Several subclasses are shown in Figure 1. For example, ‘binding reporter’ technology is used to quantify the interactions between two molecules, e.g. protein-protein, protein-small molecule, etc., while ‘inducible reporter’ technologies involve the use of a reporter gene to study the effect of perturbagens on gene expression. All of these ‘assay design’ classes have further subclasses and specification classes (Figure 1). ‘Assay design specification’ describes further details of the ‘assay design’, namely, ‘assay kit information’, ‘antibody’, ‘measured entity’, ‘signal direction’, etc. ‘Measured entity’ is a molecular entity, which is quantified as the output of a biological reaction; e.g. ‘ATP’ (adenosine triphosphate) can be quantified in a viability assay. Having the information on ‘measured entity’ in the assays allows one to analyze and optimize the type of ‘assay design’ best suited for a specific target. For example, cell viability is measured by several methods (e.g. ‘ATP content’, ‘caspase activity’, etc.) and each has a different ‘measured entity’. ‘Signal direction’ specifies whether the measured readout signal increased or decreased in the perturbed vs. the unperturbed system, which is relevant for selecting and interpreting counter and confirmatory assay results. Information about the assay kits (e.g. CellTiter-Glo®, GeneBLAzer®) provides a reference to a specific commercially available (assay kit) product and thus to analyze differences in assay sensitivity, etc. in that context.

**Figure 2:**
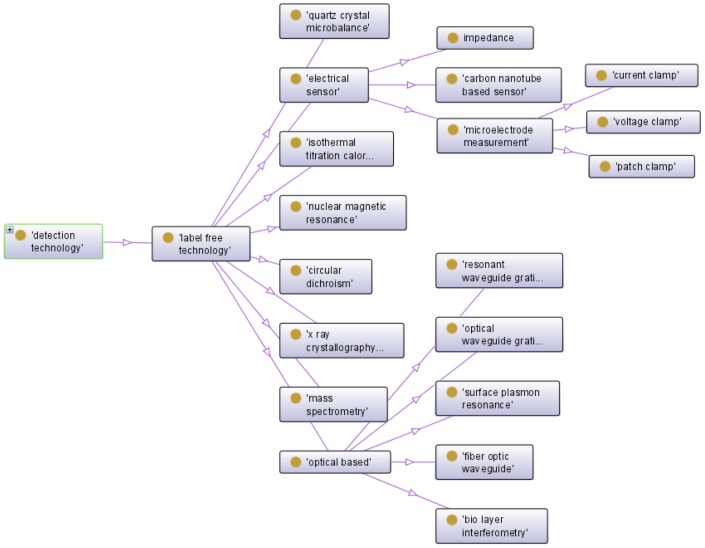
Detailed description of a subcategory of detection technology. Partial view of the class, ‘detection technology’ from BAO, which shows the sub-class, ‘label free technology’ in detail.

BAO further describes the underlying physical signal detection in bioassays as ‘detection technology’. Figure 2 illustrated one such detection technology in detail; ‘label-free technology’ includes the sub-categories ‘circular dichroism’, ‘electrical sensor’, ‘isothermal titration calorimetry’, ‘mass spectrometry’, ‘nuclear magnetic resonance’, ‘optical based’, ‘quartz crystal microbalance’, and ‘x-ray crystallography’. Some of these have further subcategories, such as ‘optical-based’ detection includes ‘bio layer interferometry’, ‘fiber optic waveguide’, ‘optical waveguide grating’, ‘resonant waveguide grating’, and ‘surface plasmon resonance’. Additional attributes of the detection technologies, such as the ‘wavelength’ at which the measurement was performed in an assay and the ‘detection instrumentation information’ (FLIPR tetra, ViewLux CCD imager) are captured in the class ‘detection technology specification’. This information is vital when comparing assay results and for inferring potential artifacts.

BAO ‘meta target’ describes the biological system, specifically the components directly perturbed by the assay. ‘Meta target’ can be directly described as a known ‘molecular target’ (e.g. a purified protein or a protein complex), or indirectly for example, by a ‘biological process’ (e.g. ‘regulation of transcription’) in which the molecular target participates and which can be described using terminology from GO [14]. Standardized descriptions of molecular or process targets enable linking external content, such as pathway databases with the goal to infer the mechanism of action of perturbagens in an assay. BAO ‘meta target’ can also be more general, for example ‘cell line’ (imported from CLO) to describe cytotoxicity or viability assays, ‘anatomical entity’ (imported from OBI), and ‘organism’ (imported from NCBITaxon) to describe organism-based assays. Additional details about targets are captured as ‘meta target specification’. Examples include ‘protein specification’ (‘protein purity’, ‘protein form’, ‘protein preparation method’), ‘cell line specification’, which includes assay-specific details about the cell line (‘cell line culturing component’, ‘cell line modification’, ‘transfection attributes’) and ‘anatomical entity specification’, which describes the ‘disposition’ of the tissue (healthy vs. diseased entity). As an example, we described the HeLa cell line used in a PubChem assay (AID 1611) in detail [38], such as its origin (organism: Homo sapiens; anatomical entity: cervix), cell type (epithelial), disease (adenocarcinoma), cell line culturing (adherent), cell line modification (stable transfection), DNA construct (heat shock promoter driven-luciferase reporter gene construct), and cell line repository (ATCC, American type culture collection).

BAO ‘endpoint’ describes how the assay results are reported following measurement of the perturbation and, in most cases, manipulations of the raw data to calculate the reported ‘endpoint’. Several ‘endpoint’ categories have been defined. In brief, ‘perturbagen concentration’ endpoints define the concentration at which the perturbagen mediates a specific response (such as ‘IC_50_’ or ‘EC_50_’). ‘Response’ endpoints report the extent/magnitude of the perturbation such as ‘percent inhibition’. ‘Protein substrate and ligand constants’ are used to express the binding interactions between labeled or unlabeled ligands with protein receptors. ‘Temperature’ type endpoints report changes in temperature as a measure of a biological reaction. Additional endpoint details are described in the ‘endpoint specification’, including ‘endpoint mode of action’, for example ‘competitive inhibition’, ‘allosteric modulation’, ‘noncompetitive inhibition’, ‘partial inhibition’, ‘tight binding inhibition’, ‘time dependent inhibition’, and ‘uncompetitive inhibition’. BAO relates the mechanisms of action to endpoint (and not perturbagen), because an assay ‘endpoint’ uniquely combines ‘perturbagen’ and assay ‘measure group’ (vide supra); and the mechanism of action is determined by the action of the ‘perturbagen’ on a specific biological component. Other endpoint attributes are ‘data manipulation specification’ (e.g. ‘curve fit specification’, ‘endpoint normalization’) and ‘unit of measurement’ (imported from UO vide infra). Knowledge about ‘endpoint’ is formalized using DL in OWL 2.0 by specifying relationships between endpoints and other BAO concepts. This enables a reasoner to infer semantic equivalences, for example, between a ‘50 percent inhibition’ endpoint measured at a defined ‘screening concentration’ and an ‘IC_50_’ value corresponding to this concentration.

**Figure 3:**
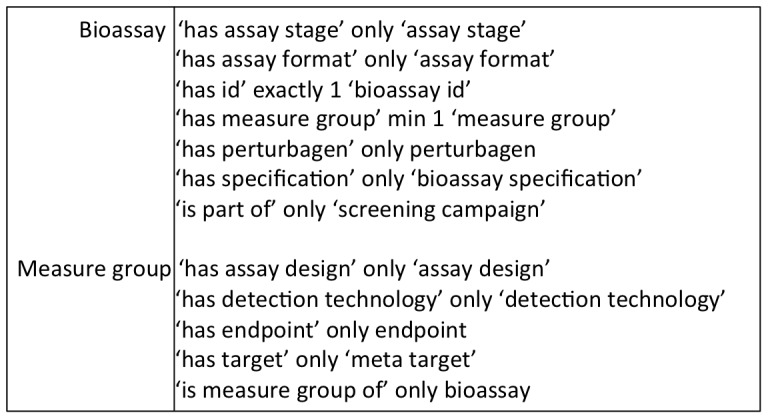
Excerpt of semantic description of a bioassay and measure group. The formal definition of a ‘bioassay’ and ‘measure group’ and the relationships that exist between them in BAO are shown.

To represent domain knowledge is one of the objectives of BAO. BAO (v1.6b1402) has SROIQ(D) [39] expressivity and consists of 1292 classes, 123 object properties, 13 data properties, and 46 individuals (not including individuals of annotated assays or endpoints). The basic definition of the concept ‘bioassay’ states that the set of objects in the domain of discourse under the interpretation of ‘bioassay’ that have at least one and only relationship, *‘has measure group’*, with the objects under the interpretation of ‘measure group’. The definition also needs to fulfill the requirement that the ‘bioassay’ objects have a unique and only identifier, *‘has id’*, defined from the data source (Figure 3). In addition, there are optional requirements that a ‘bioassay’ relates only to ‘assay stage’ through *‘has assay stage’*, ‘assay format’ through *‘has assay format’*, ‘perturbagen’ through ‘*has perturbagen*’, *‘is part of’* a ‘screening campaign’ and each ‘bioassay’ attribute settings though ‘bioassay specification’. For all the objects in the interpretation of ‘bioassay’, there exist a relationship among a set of objects represented by *‘has measure group’* and these objects must belong to the interpretation of ‘measure group’. These objects could exist in the domain without having any relationship to other objects. As an example; if there exists a relationship between this object (‘measure group’) and a set of other objects through the relationship *‘has target’*, the related set of objects must be in the interpretation of ‘meta target’. There are many ‘meta target’ objects in a ‘bioassay’: ‘molecular target’, (e.g., ‘kinase’) ‘biological process’ target, (e.g., ‘regulation of transcription’ from GO module: ‘GO_0045449’) and so on. The definition of ‘bioassay’ allows many relationships through *‘has measure group’* and *‘has target’* combinations. In addition there are relationships to the various other BAO main class hierarchies.

### Modular construction - utilizing external ontologies in BAO

**Figure 4:**
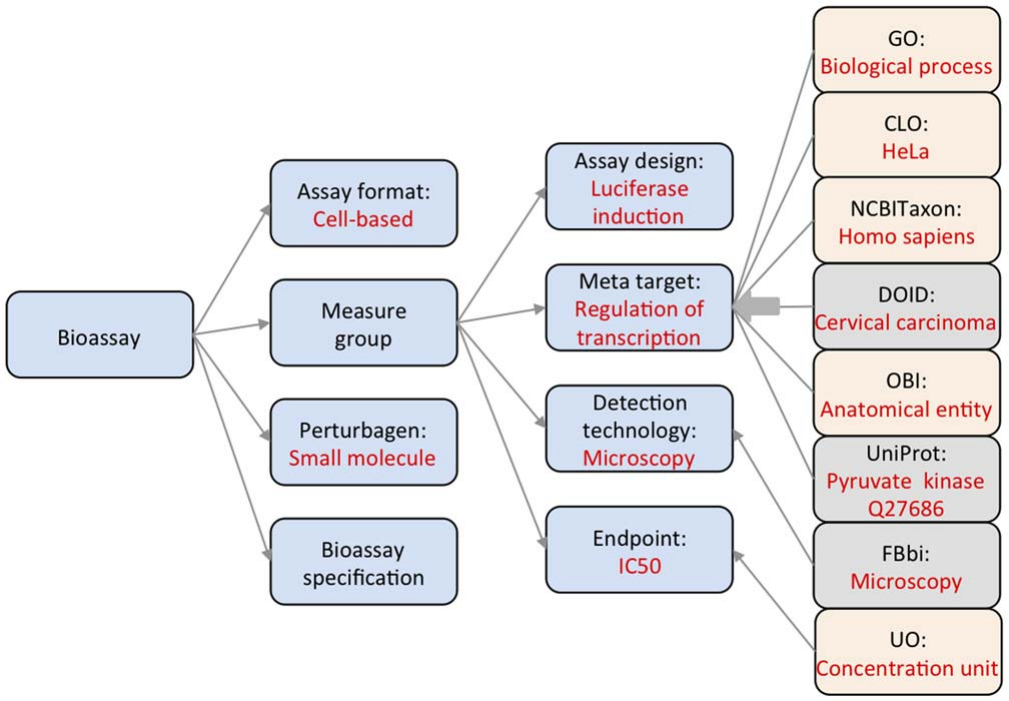
List of external resources that contribute to BAO concepts. External resources are shown to the far right and are linked to BAO concepts shown in blue to their left. The resources from which terms were already imported are shown in orange and those that will be imported in the future are shown in grey. Specific examples of terms in resource or BAO concept are shown in red letters. Human disease (DOID) and Biological imaging methods (FBbi) ontologies will be incorporated in the near future.

Several existing ontologies contain useful information to define concepts related to biological assays described by BAO. Figure 4 illustrates how these are related to BAO. Upon their analysis (vide infra) we imported relevant modules from external ontologies into BAO using OntoFox [35] and module and axiom extraction facilities built into the OWL3 API [36].

Table 1 lists the imported ontologies and the expressivity of the imported modules. From GO, the ‘biological process’ concept was imported. From CLO, the ‘cell line’ concept and cell line attributes were imported. The CLO is currently being extended as a collaborative effort to cover cell lines relevant for biological screening [38]. Organism names associated were imported from the NCBITaxon [40]. Protein target names and IDs were referenced from UniProt [41]. From units of measurement (UO), different measurement units were imported [42]. From OBI, we imported ‘anatomical entity’ [21]. BAO was not developed as an extension of OBI, because a different organization was required to facilitate categorization of assays and screening results for data retrieval and analysis. Hence, BAO was developed with a different perspective, primarily focused on querying and analysis of results, i.e. a higher level of abstraction, vs. primarily process focused. OBI links to many other resources and therefore mapping BAO to OBI may add significant value, as BAO and OBI take different, but not incompatible approaches in describing assays (vide supra). BAO can be seen as a more concise description with the specific purpose to facilitate assay annotation by specific concepts and in turn the classification and rule-based aggregation and analysis of screening data result sets [32]. This slightly more abstract nature of BAO is the reason why many relationships are very specific (connecting two BAO classes; compare BAO design above). We have mapped some of the BAO relationships to the OBO Relationship Ontology (RO) and we aim to use more RO relationships [43] in the future. We are currently working on importing the disease terminology from the Human disease (DOID) ontology, which would facilitate linking targets studied in a bioassay directly to diseases [44].

**Table 1:** List of ontologies used in BAO.

Ontology	Imported module	Expressivity of imported module
Gene Ontology (GO)	Terminology from ‘Biological process’	AL
Cell Line Ontology (CLO)	‘Permanent cell lines’ used in PubChem assays	ALE
NCBI organismal classification (NCBITaxon)	Organism names	AL
Units of measurement (UO)	Time and concentration units	AL
Information Artifact Ontology (IAO)	Information content entity	ALC
Ontology for Biomedical Investigation (OBI)	Anatomical entity	AL
OBO relationship types (OBO REL)	All of the relationships	ALIH+

### BAO Application: Standardized annotation of PubChem assays

Using BAO terms, we have manually curated and annotated 944 assays from PubChem, based on their textual descriptions (see methods). These assays correspond to over 100 million endpoint individuals (screening results), which were standardized from 299 distinct PubChem endpoint names to 20 BAO terms. Assays were categorized into campaigns consisting of (i) primary, (ii) confirmatory, (iii) secondary (counter screening, selectivity, MMOA (molecular mechanism of action) characterization, etc.), (iv) lead optimization stages, and (v) summary (a PubChem specific term) assays. In PubChem, related assays belonging to one screening campaign are listed in a summary bioassay; however, the list is not always comprehensive and the organization of assays into campaigns therefore requires manual curation. We manually identified and included those assays missing from each campaign and created relationships among them using the object property *‘is related assay to’* and its sub-types *‘is confirmatory assay of’*, *‘is counter assay of’*, *‘is identical assay to’*, *‘uses orthogonal technology to’*, etc., as defined in BAO. This facilitates rule-based aggregation of data by screening campaigns and also analysis of assay results across campaigns.

**Figure 5:**
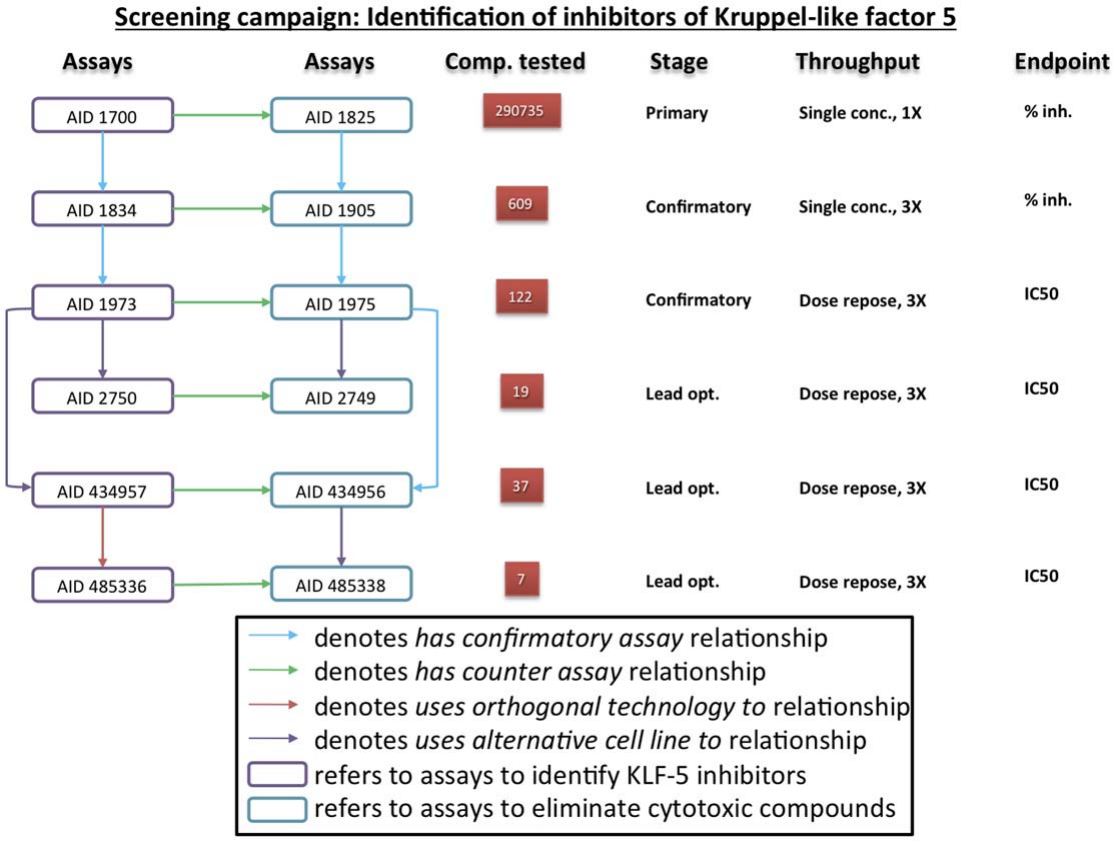
Formal BAO annotation of a PubChem screening campaign. In the campaign to identify inhibitors of Kruppel-like factor 5, the investigators screened compounds, both to identify inhibitors of KLF5 and to eliminate cytotoxic compounds. The compounds tested at each stage are shown in red boxes. The endpoints (result type) from each of the assays are listed to the right. Abbreviations: Comp: compounds, opt: optimization, conc: concentration, inh: inhibition, 1×: compound tested in singlicate, 3×: compound tested in triplicate, and CID: compound ID.

Here we illustrate annotations of a PubChem assay (AID 1700) using BAO terminology. In this campaign, the investigators are attempting to identify small molecule inhibitors of Krüppel-like factor 5 (KLF5), as studies showed that KLF5 inhibition reduces proliferation of human colorectal cancer cells and intestinal tumor formation in mice [45], [46]. They screened small molecules in parallel for both identifying inhibitors of KLF5 and eliminating unwanted cytotoxic compounds (Figure 5). To accomplish this, compounds were screened in two primary HTS assays followed by corresponding confirmatory (triplicate) assays and subsequent further confirmation by concentration-response screening. The two parallel screening lines (Figure 5) served as counter assays to remove undesired hits (cytotoxic compounds). The identified and prioritized actives were moved forward into lead optimization (AIDs 2750, 2749, 434957, 434956, 485336 and 485338). The ‘screening campaign name’ was annotated as “Identification of inhibitors of Krüppel-like factor 5” and the PubChem assigned ‘assay title’ is “Primary cell-based high throughput screening assay to identify inhibitors of Krüppel-like factor 5 (KLF5)”. This assay was annotated with the main BAO categories as follows: ‘assay format’ is ‘cell-based format’; ‘assay design’ is ‘inducible reporter: luciferase induction’; ‘meta target’ is ‘biological process’: ‘regulation of transcription’, with the ‘transcription factor’ being Krüppel-like factor 5′ and ‘permanent cell line’ is ‘DLD-1’; ‘perturbagen’ is ‘small molecule’; ‘detection technology’ is ‘luminescence’; and ‘endpoint’ is ‘response endpoint’: ‘percent response’: ‘percent inhibition’. All BAO classes correspond to unique BAO IDs (not shown). Additional details about the main annotations were captured following the ‘bioassay component specification’ class hierarchy, including ‘format specification’: ‘assay phase characteristic’ which is ‘heterogeneous assay’; ‘assay design specification’: ‘measured entity’ which is ‘luciferase’; ‘meta target specifications’ include ‘cell line modification’: ‘stable transfection’, and ‘DNA construct’: ‘luciferase gene with Krüppel-like factor 5-promoter’. ‘Perturbagen specification’ includes ‘perturbagen screening concentration’ which is 5 micromolar. ‘Design specification’ includes ‘signal direction’: ‘signal decrease’. ‘Endpoint specification’ includes ‘endpoint mode of action’, which is ‘inhibition’. Several assay attributes were captured via the subclasses, ‘bioassay specifications’, which include ‘bioassay type’: ‘functional’, ‘assay stage’: ‘primary’, ‘assay measurement throughout quality’: ‘single concentration single measurement’. The ‘assay source’ is ‘The Scripps Research Institute Molecular Screening Center’. The ‘compound library’ is ‘MLSMR (Molecular Libraries Small Molecule Repository)’. The context of the assays in the entire screening campaign is captured via the assays relationship as follows: AID 1700 *‘has confirmatory assay’* 1834; *‘has counter assay’* 1825 and *‘has summary assay’* 1858. During the lead optimization stage, some of the assays were performed in a colorectal cancer cell line, DLD-1, instead of IEC-6, which was derived from healthy tissue. These assays have the *‘uses alternative cell line to’* relationship. The assay relationships, number of compounds tested, the ‘assay stage’, ‘assay measurement throughput quality’ and ‘endpoint’ are shown in Figure 5. This campaign resulted in the identification of two active compounds, namely, CID 5951923 and CID 439501. Results from this screening campaign have recently been published [47].

### Statistical analysis of PubChem assays and screening campaigns by BAO concepts and relationships

**Figure 6:**
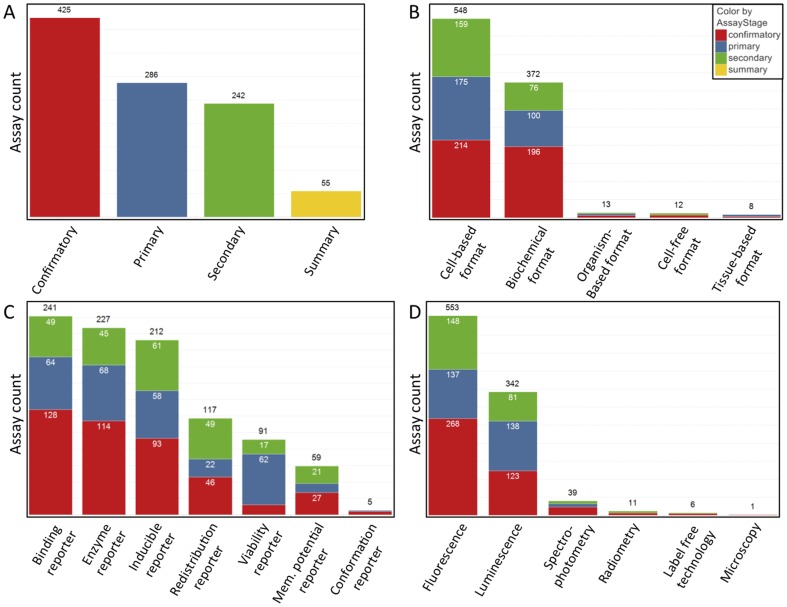
Summary of PubChem assay annotations. Statistics of 944 curated PubChem assay by the BAO main classes ‘assay stage’ (A) ‘assay format’ (B), ‘assay design’ (C) and ‘detection technology’ (D).

Using BAO annotations, which are organized in several hierarchies, assays can be readily categorized with varying levels of granularity. We performed statistics on 944 PubChem assays that were manually curated. Categorizations of assays based on the subclasses of ‘bioassay specification’: ‘assay stage’ included 286 ‘primary’, 425 ‘confirmatory’, 242 ‘secondary’, and 55 ‘summary’ assays (Figure 6A). In terms of assay ‘format’, the majority of assays were categorized as ‘cell-based format’ (548) or ‘biochemical format’ (372), which means that they were either annotated directly as one of these classes or as a subclass within the respective sub-hierarchies. There were also a few assays having ‘organism-based format’ (13), ‘cell-free format’ (12), and ‘tissue-based format’ (8) (Figure 6B). The ‘assay design’ was curated as ‘binding reporter’ (241), ‘enzyme reporter’ (227), ‘inducible reporter’ (212), ‘redistribution reporter’ (117), ‘viability reporter’ (91) and a few ‘membrane potential reporter’ (59) and ‘conformation reporter’ (5) types (Figure 6C). Assays were annotated with the following ‘detection technologies’: ‘fluorescence’ (553), ‘luminescence’ (342), ‘spectrophotometry’ (39), ‘radiometry’ (11), ‘label free technology’ (6), and ‘microscopy’ (1) (Figure 6D). Based on the property or processes that the assay was interrogating, the annotated PubChem ‘bioassay type’ was as follows: ‘functional’ (722), ‘binding’ (193), and ‘ADMET’ (absorption, distribution, metabolism, excretion and toxicity, 33). This information is relevant to interpret compound activity, for example, biochemical assays provide direct evidence of the mechanism of action (e.g. inhibition of an enzyme) while activity in cell-based assays also implies that a compound is cell permeable.

Standardized annotations of the BAO class ‘endpoint’ (compare Figure 1) among the 944 curated assays are shown in Table 2.

**Table 2:** Summary of the standardized ‘endpoint’ in PubChem assay annotations.

Endpoint standardized	Count
Binding constants: Bmax	9
Binding constants: Kd	13
Endpoint: Perturbagen concentration	1
Endpoint: Response	3
Fold response: Fold activation	16
Fold response: Fold inhibition	1
Percent response: Percent activation	70
Percent response: Percent growth inhibition	2
Percent response: Percent inhibition	171
Perturbagen concentration: AC50	208
Perturbagen concentration: CC50	5
Perturbagen concentration: EC50	155
Perturbagen concentration: IC50	238
Perturbagen protein affinity: Ki	8
Response: Maximal response	184
Response: Percent response	27
Response: Percent viability	16
Response: Raw activity	41
Temperature: Tm	3

We have previously demonstrated the utility of categorizing assays by design and technology to identify assay artifacts and to infer their mechanism of action [32]. BAO enables the analyses of assays results in the context of any of its subsumption trees and other relationships. For example, BAO can be applied to analyze the influence of important differences in the assay conditions, e.g., the presence of ‘detergent’, ‘reducing agent’, etc. In PubChem AIDs 584, 585, 1002 and 1003, compounds were screened to identify AmpC beta-lactamase inhibitors, both in the presence and absence of 0.01% triton X-100. These assays were performed at NCGC, where they used the aggregation profiling approach to identify sensitivity of aggregate formation of compounds to detergent [48], [49]. In another campaign, the investigators screened for inhibitors of caspase-1 (AIDs 888, 929 and 996) and used reagents with different redox potential (dithiothreitol, cysteine, or catalase) to eliminate false positives that could result from compound-generated reactive oxygen species [50], [51]. In our PubChem assay annotations, we capture the special reagents added in an assay; facilitating the analysis of the effects of those reagents on compound activity.

**Figure 7:**
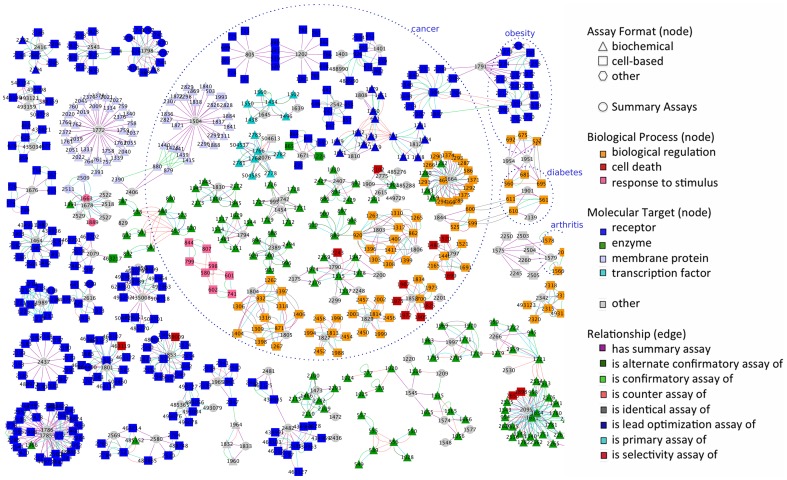
Network visualization of PubChem assays (nodes) connected by BAO assay relationships (edges) to describe screening campaigns. Shown are 682 assays that are each part of a set of at least 7 connected assays comprising 85 campaigns. Assays are identified by their AID. BAO assay annotations are also shown, including, assay format (node shape), assay target main class (node color), and BAO assay relationships (edge color). Screening campaign-disease associations were obtained from the assay descriptions (shown as areas surrounded by dotted lines).

In total, we annotated 212 campaigns from PubChem. In Figure 7, we visualized how assays are connected by the various BAO relationships to describe a screening campaign. Into this analysis we included 682 assays that are each part of a sub-network of at least 7 connected assays and which form 85 distinct screening campaigns. Figure 7 also illustrates assay format (node shape), assay target main class (node color), assay relationship (edge color) and disease association (areas surrounded by dotted line), which were obtained from the assay descriptions. Different campaigns can be connected, for example by the *‘is identical assay of’* relationship (42 such relationships are shown) connecting two (identical) assays that are part of separate projects. Most relationships describe how assays are related within one campaign/project; they include: *‘has summary assay’* (602), *‘is alternated confirmatory assay of’* (56), *‘is confirmatory assay of’* (223), *‘is counter assay of’* (201), *‘is lead optimization assay of’* (7), *‘is primary assay of’* (456) and *‘is selectivity assay of’* (18), with the counts shown in parentheses. Among the assays shown in Figure 7, assay formats are annotated as follows (only the main categories are shown in the figure): ‘biochemical format’ (220), ‘cell-based format’ (352), ‘cell-free format’ (6), ‘organism-based format’ (1), and ‘tissue-based format’ (8); molecular targets include the following categories: ‘receptor’ (264), ‘enzyme’ (127), ‘membrane protein’ (68), ‘transcription factor’ (14), ‘secreted protein’ (6), and ‘cytosolic protein’ (3); the biological process target (GO) categories include: ‘biological regulation’ (80), ‘cell death’ (14), ‘response to stimulus’ (10), and ‘cell proliferation’ (1). There are 95 summary assays (displayed as grey circles) that contain the summary information from each campaign in PubChem (vide supra).

### Excel-based BioAssay annotation template

To facilitate reporting of assays and screening results using standardized terms from BAO, we developed a user-friendly annotation tool called BioAssay Annotation Template (BAT) [52]. The Excel Annotation Template work sheet includes the metadata field names to be annotated with the corresponding definitions/descriptions of the fields. It includes general assay information and is organized by the main BAO categories. For many of the annotation fields, the template specifies a range of allowed terms from BAO, which are presented as a drop-down list/menu. BAT also defines the scope of annotations (minimum information) needed to describe an assay.

**Figure 8:**
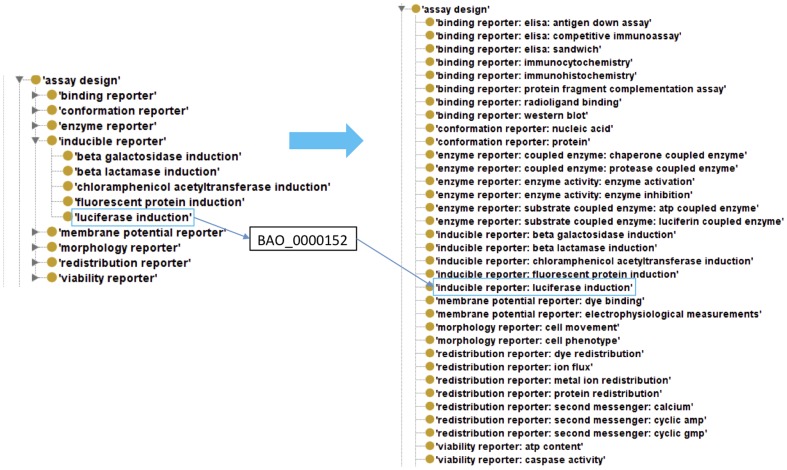
Generation of a hierarchy-flattened BAO format. The hierarchy-flattened format of BAO contains only the most specific leaf nodes. Lead node IDs were maintained in this process. The labels/names in the flattened version of BAO reflect the class hierarchy in BAO.

The creation of the assay annotation template is described in the methods section and illustrated in Figure 8. The hierarchy-flattened version of BAO used to create BAT includes all the leaf nodes of BAO with a label/name that reflects the original BAO subsumption hierarchy (starting at the major sub-hierarchies). Importantly, the BAO IDs of the leaf nodes are retained, i.e. each ID of a term in the flattened version of BAO is identical to the corresponding ID of the original BAO leaf node. BAO is version controlled and each annotation template corresponds to a specific BAO version. It is thus easy to map annotations from a previous BAO/BAT version to a later one (for example, if a label changes). Because assay annotations using BAT correspond to BAO class IDs, it is possible to leverage the ontology for querying and analysis of annotated assays, including inference by reasoning. In a simple example, BAO defines several sub-categories of ‘inducible reporter’, which can be annotated with BAT. This allows for implementation of functionality to return all subcategories for a concept query such as ‘inducible reporter’, as implemented in BAOSearch, our semantic search software application based on BAO [53]. It also allows categorization of assays based on a higher level in the hierarchy (such as ‘assay design’ as described above), based on the more descriptive annotations (via subsumption reasoning); this is also implemented in BAOSearch. A recent version of BAT can be downloaded from our website [34].

## Discussion

Large amounts of HTS data are generated in private and public organizations. Nevertheless, large-scale screening capabilities have so far not translated to increased numbers of approved drugs [54]. One of the reasons is that it is difficult to efficiently derive knowledge from increasing amounts of data, which requires distributed data integration and sophisticated meta-analyses [10]. This is currently hindered by a lack of standards and semantics in the description of screening experiments and outcomes. As a consequence, currently the enormous small molecule screening datasets cannot be utilized to their full potential to inform drug discovery and drug repositioning efforts and to advance our understanding of polypharmacology and promiscuity of compounds. The BioAssay Ontology (BAO) has been developed to address this challenge. BAO formally describes biological screening assays by numerous concepts that are relevant to categorize, analyze, and integrate large and diverse HTS datasets. BAO provides a framework for researchers to annotate high throughput screening assays. BAO was developed with the primary focus on facilitating data integration and analysis applications; it therefore has different structure and abstraction level than process-focused descriptions. To enable assay annotation, we have developed an Excel-based BAO annotation template (BAT). This template was evaluated by the MLPCN to annotate various assays in PubChem with the most relevant BAO classes. These annotated assays are available in PubChem [55]. In addition, we are currently working with the MLPCN to use BAO to annotate all MLPCN PubChem assays (>4,000) and make them accessible as a catalog of assay protocols.

The main purpose of BAO is to enable researchers to leverage the aggregated corpus of publically available data to better understand the molecular mechanisms of action of perturbagens in biological model systems. This will aid in developing and testing hypotheses related to the interplay of molecular biological components and how their perturbation influences biological function. BAO addresses 1) the development of standardized terminology and uniform standards to report HTS results; and 2) a semantic description of bioassays and their results to model domain knowledge and to facilitate semantic integration with other resources [56], [57] and to enable contextual analysis and interpretation of results. We have already used BAO to annotate a large number of PubChem assays and demonstrated that BAO concepts are useful to categorize and analyze screening results [32]. We have illustrated the use of DL to incorporate semantics into BAO concepts and to retrieve inferred query results [37]. BioAssay Ontology enables scientists to retrieve, analyze and compare diverse biological datasets from PubChem, thus accelerating the identification and prioritization of chemicals with a desired MMOA.

**Figure 9:**
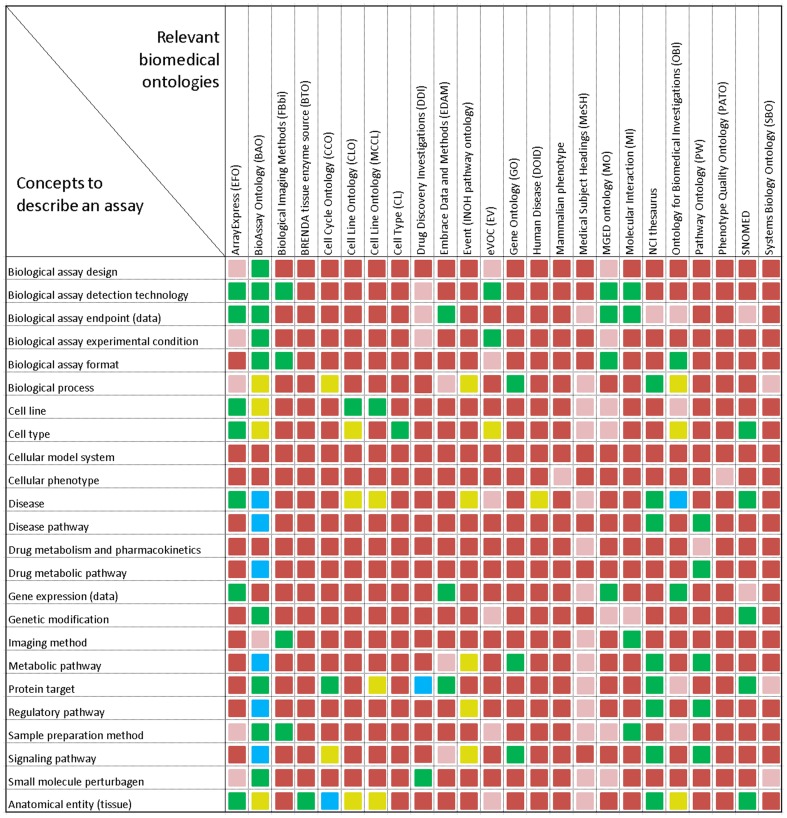
Analysis of bio-ontologies to describe chemical biology HTS assays. Coverage of biomedical concepts/terms to describe HTS assays (shown as rows) by various existing biomedical ontologies (shown as columns) is depicted. The color codes are as follows: green: the concept is well described by the ontology, pink: the concept is partially described, red: no (or little) information is available in the ontology, yellow: the concept is imported from an external reference/ontology, blue: the ontology only includes a placeholder to an external reference of that concept.

We performed a systematic comparison of BAO to other relevant biomedical ontologies, with respect to their coverage of domain knowledge required for describing chemical biology HTS assays of the type in PubChem (Figure 9). In contrast to any of the investigated ontologies, BAO well describes the relevant concepts of chemical biology screening, for example, biochemical and cell-based assays, a variety of technologies and assay designs, different types of biochemical and cellular targets, commonly reported screening endpoints, as well as various assay metadata to describe the experimental and discovery context. Existing ontologies lack many of the concepts required to model HTS data, such as assay design, detection technologies, standardized endpoints, HTS platforms, and detailed bioassay specifications. However, some concepts are well defined in existing bio-ontologies. Examples include, biological process (GO), cell line (CLO), cell type (CL), taxonomy (NCBITaxon). Thus, in the development of BAO, we leveraged those pieces to avoid duplication of work (vide supra). For some concepts, such as cellular phenotype, cellular model system, drug metabolism, and pharmacokinetics, there is currently no satisfactory reference, and therefore exists a need to develop these resources for the research community.

BAO remains under active development. The current effort is primarily focused on incorporating and aligning content from several other resources, including ontologies and data repositories. This gives us the potential to make BAO-annotated HTS data widely accessible via a “Linked Data” approach [58]. We plan to expand BAO using Semantic Web software tools to harness the enormous amount of publically available chemical probe data and to integrate these datasets with several orthogonal bioinformatics resources, including biological pathways and processes, and cellular model systems and phenotypes. Various studies associate known protein binders (e.g. activators or inhibitors) with biological functions via pathways (signaling, regulatory, metabolic) [59]–[61]. These approaches require knowledge of the target (typically from biochemical screening results). However, cell-based and increasingly image-based phenotypic assays with unknown targets constitute the majority of available data [62]. The NIH LINCS program generates molecular signatures across a wide variety of cell types using various high-throughput screening approaches [4]. The development of data standards is an important component of this project and BAO is being applied to describe these data [63]. Pathways will be integrated via the BAO class ‘meta target’. To interpret, integrate, and analyze cell-based screening results, the relevant components and properties of the cellular systems will be formalized into a knowledge model. We are collaborating with the CLO group to extend the CLO [64] towards cell lines and primary cells, which are typically used in cell-based assays [38].

The goal of BAO and BAT is to provide a community resource and a standard to annotate and describe screening assays and results. Based on requirements and suggestions from the HTS and chemical biology communities, BAO will be extended and optimized. It should be noted though that ontology development is largely a manual effort and requires significant resources in terms of domain expertise and knowledge formalization. New versions will be uploaded to the NCBO BioPortal and the BAO website. With each new BAO version, an updated BAT can be generated.

BAO is freely available and under active development. For the most current release and a wide variety of information related to the BAO project we refer to our website and Wiki [23]. We continue to annotate assays and develop software tools related to BAO. Our BAOSearch Semantic Web application makes it very easy to query, search, explore, and download BAO-annotated assays, standardized screening results, and chemical structures, and is freely available [65].

### Summary and Conclusions

BAO describes biological assays and their outcomes by concepts that are relevant to interpret, integrate, aggregate, and analyze screening data. BAO addresses 1) development of controlled terminology and uniform standards to report HTS and lower throughput probe and drug discovery assays and results; and 2) a semantic description of bioassays and their results to formalize domain knowledge and to facilitate semantic integration with diverse other resources [14], [38], [40], [42]. We have used BAO to annotate PubChem assays, provided statistics and showed that BAO concepts are useful to categorize and analyze screening results [32]. Beyond individual assays and results, BAO also includes relationships to describe screening campaigns. We have illustrated how various BAO (inter-assay) relationships connect 682 assays into 85 screening campaigns. BAO facilitates identification of important trends, such as assay artifacts or preferred technologies. BAO can also be used to develop hypotheses about the mechanism of action of perturbagens. We are developing BAO into a community standard to describe assays and their results with the goal to enable the integration of diverse datasets and to facilitate the interpretation and global comparison and analysis of assay experiments and screening outcomes. BAO also includes information to enable linking external content, such as pathway databases. Thus, BAO opens a new approach to formally describe and analyze HTS datasets with potential to discover new knowledge by inference.
